# “Life on the line”: leiomyosarcoma causes right ventricular inflow tract obstruction

**DOI:** 10.1007/s10396-022-01280-w

**Published:** 2023-01-11

**Authors:** Xuejie Li, Xuewei Liu, Ronghua Zhou

**Affiliations:** Department of Anesthesiology, Chinese Academy of Medical Sciences, West China Hospital, Sichuan University and The Research Units of West China (2018RU012), Chengdu, 610041 Sichuan China

**Keywords:** Leiomyosarcoma, Right ventricular inflow tract obstruction, Transesophageal echocardiography

**Fig. 1:**
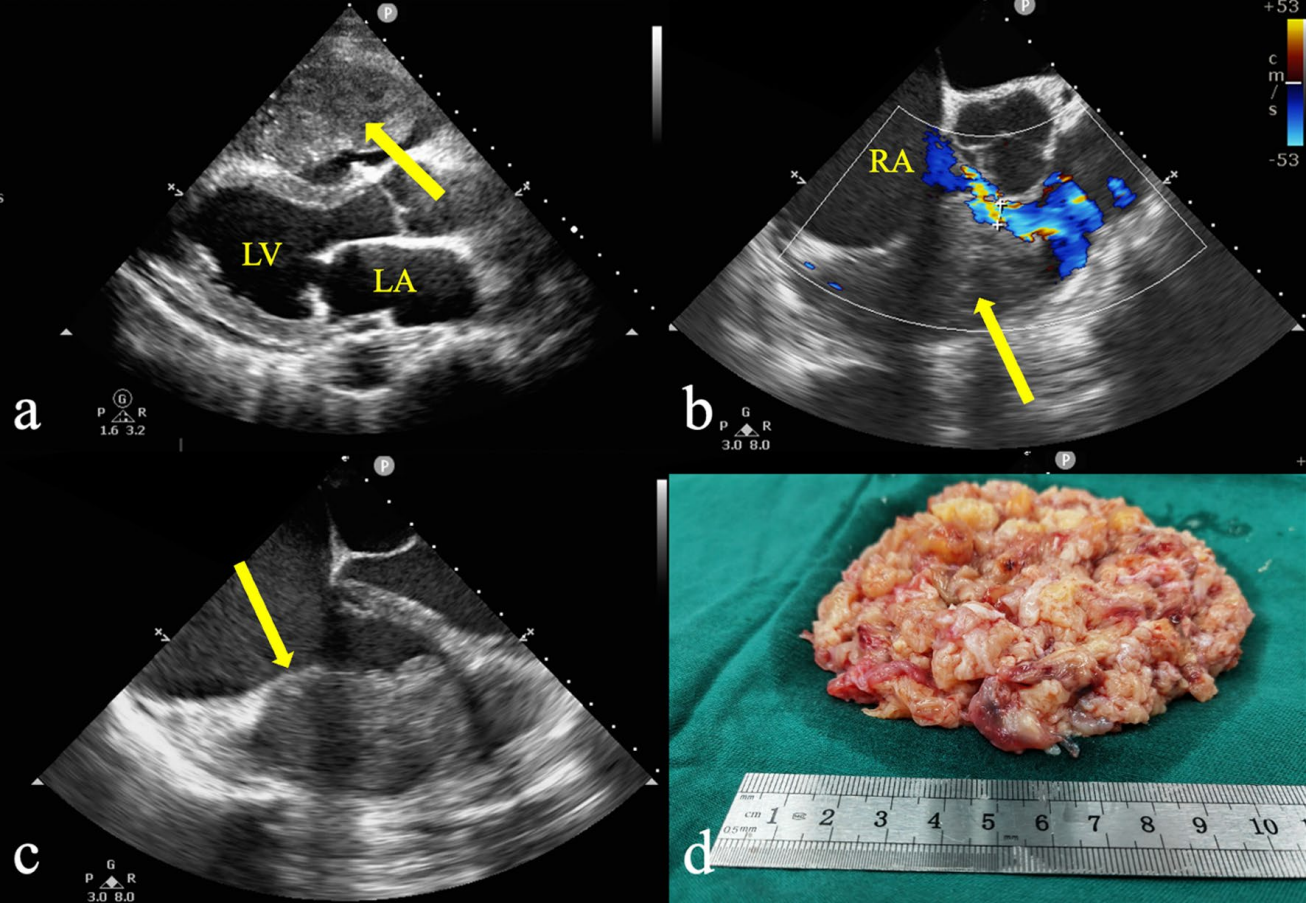
**a** Transthoracic echocardiography showed a huge mass (yellow arrow) occupying the inflow tract and most of the right ventricular cavity. **b** In the mid-esophageal RV inflow–outflow view, the mass was closely related to the right ventricular free wall (yellow arrow) and the patient had only a narrow gap of approximately 6 mm between the tumor and the supraventricular crest to provide forward blood flow during diastole. **c** The mass fused and wrapped with the anterior and posterior tricuspid valves, and the demarcation between the tumor and the myocardium was not clear (yellow arrow). **d** The excised fragmented tumor tissue. The findings of the pathological examination suggested leiomyosarcoma. *LA* left atrium, *LV* left ventricle, *RA* right atrium

A 43-year-old man was admitted to the emergency department with symptoms of lower extremity edema and decreased activity tolerance with progressive dyspnea. Echocardiography showed right ventricular inflow tract obstruction due to a massive right ventricular mass (size: 90 mm × 65 mm × 52 mm). The right ventricle was almost completely occupied by the mass (Fig. 1a). Urgent resection of the right ventricular mass was planned. In the RV inflow–outflow view, the patient had a narrow gap of approximately 6 mm between the mass and supraventricular crest (Fig. 1b). Since the angle of blood flow and the sampling line were perpendicular, Doppler echocardiography was not suitable for the detection of blood flow velocity. Fusion and wrapping of the anterior and posterior tricuspid valves were observed and only the septal valve was active (Fig. 1c). The supraventricular crest of the right ventricle is a rounded accentuation of its muscular wall that demarcates the inflow and outflow tracts [1]. The tumor mainly occupied the space from the tricuspid valve to the supraventricular crest causing obstruction of the inflow tract. Median thoracotomy under cardiopulmonary bypass was urgently performed. The anterior and posterior valves of the tricuspid valve were completely invaded by the mass and were difficult to separate. Tricuspid bioprosthetic valve replacement was performed. The mass occupied the entire right ventricle and infiltrated the endocardium and myocardium and could not be completely resected because of the extensive local invasion. An image of the excised fragmented tumor tissue is shown in Fig. 1d.

With a frequency of 0.02%, cardiac tumors are very rare neoplasms, and only 25% of them are malignant [2]. The most common histological type of primary cardiac tumor is angiosarcoma, and leiomyosarcomas appear in < 1% of the cases [3]. The tumor in this patient was pathologically diagnosed as leiomyosarcoma. TEE provided a guide for surgical decision-making and evaluation of the hemodynamics and cardiac function, demonstrating two important findings. First, the anterior and septal valves of the tricuspid valve were eroded by the tumor, indicating that tricuspid valve replacement would be necessary if tumor resection was to be performed. Second, the evaluation of right heart function is also a very important issue. The RV FAC of the patient was 18% after anesthesia, 13% on the 1st day after surgery and 15% on the 8th after surgery, suggesting persistent RV dysfunction.

We experienced an extremely rare emergency case of right ventricular inflow tract obstruction caused by primary cardiac leiomyosarcoma. TEE played a crucial role in surgical decision-making and anesthesia management.
